# The Combined and Comparative Impacts of Financial Incentives Versus Practice Facilitation Implementation Support for Social Risk Screening in Community Health Centers

**DOI:** 10.1111/1475-6773.14448

**Published:** 2025-02-10

**Authors:** Danielle Hessler, Miguel Marino, Jorge Kaufmann, Rachel Gold, Anne King, Holly Wing, Jenna Donovan, Maura Pisciotta, Sara Ackerman, Bruce Goldberg, Laura M. Gottlieb

**Affiliations:** ^1^ University of California San Francisco California USA; ^2^ Oregon Health and Science University Portland Oregon USA; ^3^ OCHIN Inc. Portland, Oregon USA; ^4^ Kaiser Permanente Center for Health Research Portland Oregon USA; ^5^ Oregon Rural Practice‐Based Research Network Oregon Health and Science University Portland Oregon USA

**Keywords:** community health centers, financial incentives, practice facilitation, social determinants of health, social risk screening

## Abstract

**Objective:**

To examine the impact of two interventions aimed at increasing the adoption of social risk screening in community health centers (CHCs).

**Study Setting and Design:**

Intervention CHCs were in one of three groups, which received either: (1) tailored practice facilitation‐focused social risk screening implementation supports; (2) financial incentives for screening; and (3) both practice facilitation and financial incentives in staggered order. A group of control clinics was identified through propensity score matching and a difference‐in‐difference analysis compared effects across groups.

**Data Sources and Analytic Sample:**

Using electronic health record data, we calculated monthly rates of social risk screening (per 100 adult patients) at 32 intervention clinics (19 practice facilitation supports only, 6 financial incentives only, 7 both financial incentives and practice facilitation supports), and 32 control clinics.

**Principal Findings:**

Compared to control clinics, clinics in any intervention group had a greater increase in average monthly social risk screenings from pre‐ to post‐intervention that was maintained over the 24 months following intervention (difference‐in‐difference: 4.66, 95% CI: 0.89, 8.43). In the primary analysis, clinics engaged in both interventions increased screening rates when practice facilitation implementation supports were added to financial incentives (12 months 3.70, 95% CI: 0.34, 7.07; 24 months 4.18, 95% CI: −0.01, 8.87); adding financial incentives to practice facilitation supports resulted in increased screening rates but did not reach statistical significance.

**Conclusions:**

This study is the first to compare different interventions intended to bolster CHCs' social risk screening activities. As social risk screening becomes increasingly tied to US policy and payment structures, it is critical to identify strategies that can support implementation in settings serving underserved populations. Our findings suggest modest impacts of both financial incentives and practice facilitation supports.

**Summary**

- What is known on this topic
  ○ Social risk screening is increasingly tied to US policy and payment, yet little is known about how to best support community health centers (CHCs) to implement social risk screening.
- What this study adds
  ○ We found modest impacts of financial incentives and practice facilitation supports in CHCs. Clinics receiving these implementation interventions screened four to five more patients per 100 patients per month than nonintervention sites.

## 1 Introduction

The increasing recognition that health and health equity are powerfully shaped by social and economic environments [1, 2, 3, 4] has led multiple federal, state, and local agencies to recommend, and in some circumstances require, that healthcare providers identify and address patients' social risks (e.g., food insecurity, housing instability, transportation needs) as part of high‐quality care [5, 6, 7, 8, 9]. These activities are increasingly tied to payment structures [10]. As a result, new social risk screening tools have emerged to support healthcare‐based social risk assessments. Leading electronic health record (EHR) vendors now include social risk screening tools in their core products, and several web‐based interfaces help users to identify and support referrals to relevant community organizations. These tools have the potential to be especially impactful in community health centers (CHCs), which provide care to > 26 million patients annually and disproportionately serve patient populations most at risk for the negative health outcomes associated with social and economic disadvantage [11]. Despite these new policies and resources, little is known about how to maximize the implementation and sustainability of social risk screening and community referral programs while accounting for limited clinical resources and the potential burden of these programs on clinic staff.

The existing literature on strategies to facilitate any clinical practice changes points to important differences—and synergies—in the effectiveness of hands‐on practice coaching/facilitation and monetary (e.g., providing incentive payments to clinics based on rates of completion) approaches [12, 13, 14, 15, 16]. Though both are known to affect the uptake of specific activities in select contexts [13, 14, 17, 18], few studies have examined the impact of these strategies on social risk screening in clinical practices [19]. One exception to this is the ASCEND trial, which reported that social risk screening was 2.45 times (95% CI: 1.32, 4.39) higher over the 6 months clinics received practice facilitation implementation support compared with the pre‐intervention period but diminished during the post‐implementation support period (rate ratio, 2.16; 95% CI: 0.64, 7.27) [19]. To our knowledge, no studies have compared the impacts of different approaches on social risk screening rates. Since clinics seeking to meet new screening requirements will have to implement these practice changes, understanding the combined and comparative impacts of practice facilitation and financial incentive implementation supports is critical.

We leveraged the unique confluence of two recent efforts that aimed to increase the adoption of social risk screening in CHCs. First, in ASCEND, a federally funded pragmatic stepped‐wedge trial, participating CHCs were provided 6 months of tailored social risk screening practice facilitation implementation supports that included technical support to use the EHR to document screening results [19].

Second, the Accountable Health Communities (AHCs) demonstration project supported social risk screening and social service referrals in 31 communities across the United States [9, 20] The Oregon AHC site received approval from the Centers for Medicaid and Medicare Services (CMS) to provide financial incentives to participating clinics, meaning clinics were reimbursed for each social screening conducted. Clinics also received light technical assistance to help staff use an electronic database outside of the EHR for documenting screening response [21]. Select Oregon CHCs engaged in both initiatives. The naturally occurring overlap of these two large implementation efforts, both in terms of timing of the two initiatives and participating clinics, created a unique natural experiment opportunity to evaluate how financial incentives and practice facilitation implementation strategies can independently and in combination affect the uptake and sustainability of social risk screening in CHCs.

In this study, we examined social risk screening rates both while clinics were receiving supports and following receipt of supports and compared results from intervention groups to a propensity matched set of controls. We asked two questions: (1) what is the impact of CHC clinic engagement in any implementation support program (AHC financial incentives and/or ASCEND practice facilitation support) on social risk screening rates compared to control clinics and (2) what is the added benefit of combining financial incentives and tailored practice facilitation implementation supports versus providing either alone? We hypothesized that any form of implementation support would benefit CHC engagement in social risk screening when compared to no supports, and that combining monetary and practice facilitation supports would have even greater impact.

## 2 Methods

### 2.1 Study Design

We leveraged a natural experiment design to examine the impact of two distinct interventions aimed at increasing the adoption of social risk screening rates in CHCs, each beginning in 2018. In both ASCEND and AHC, the intervention employed a staggered role out to multiple clinics. Moreover, some clinics participated in both ASCEND and AHC. This naturally occurring overlap in implementation efforts allowed us to compare financial incentives versus practice facilitation implementation supports and to assess the benefit of combining the two strategies.

### 2.2 Interventions

The ASCEND CHCs received 6 months of practice facilitation and technical assistance in the use of relevant EHR tools to support social risk screening within a shared Epic EHR and how to use these tools in clinic workflows, both tailored to the individual clinic's needs [19, 22]. Intervention details have been described in detail previously [22]. In brief, the trainer/coach team guided study clinics though a five‐step implementation process: secure leadership buy‐in; set goals; develop workflows; orient staff and implement and iterate. Throughout the 6‐month intervention, the trainer‐coach team met with clinic representatives two to three times monthly to support progress. The CHC clinics participating in the Oregon demonstration of the AHCs program received a web‐based data tool to document social risk screening with light technical assistance in the form of one to two meetings specifically focused on supporting tool use and related workflows (e.g., consultants helped navigate technical problems related to tool use, and tool use workflows within clinic), as well as a financial reimbursement paid to the clinic on a per screening basis for Medicaid and Medicare patients ($10 per completed social risk screening in the first 12 months of implementation, followed by a reduction to $2 per screen in Months 13–36) [21].

### 2.3 Data Source and Participants

OCHIN Inc. provides a fully hosted and shared EHR platform, OCHIN Epic, which in 2018 was shared by 593 independent clinic sites providing community‐based care in 16 US states. Patient‐level data from discrete fields in this EHR, aggregated by clinic and by month, were used in these analyses. The current study was comprised of 64 OCHIN‐member primary care clinic sites in total. The ASCEND trial consisted of 26 OCHIN member sites that received social risk screening implementation supports; its study protocol, including clinic recruitment, and study results have been published previously [19]. Seven of the 26 ASCEND participants were also part of AHC. Another six OCHIN‐member sites participated in AHC alone. These 32 clinics (19 ASCEND‐only, 6 AHC‐only, and 7 dual) comprised the total intervention sample of the current study and were located in California, Georgia, Massachusetts, Montana, Ohio, Oregon, Washington, and Wisconsin. Utilizing nearest neighbor matching with a set of baseline covariates we identified 32 control sites located in California, Massachusetts, Ohio, Oregon, Washington, and Wisconsin. Covariates and matching process are described below.

### 2.4 Study Period

We selected a study period of March 2018 to April 2023 to allow for a 6‐month baseline period prior to the first intervention start and at least 18 months of post‐intervention data for each clinic. The first intervention began September 2018 and the last began February 2021, totaling 13 distinct start times. More specifically, clinics participating in ASCEND started the intervention between September 2018 and July 2021, while clinics participating in AHC started that intervention between December 2018 and April 2019. Each start time included between one and five intervention sites constituting 13 group‐time combinations for use in Callaway and Sant'Anna difference‐in‐difference (CS‐DID). Of note for considering the COVID‐19 pandemic, all six AHC‐only sites began intervention nearly a year prior March 2020, while 12 of the 19 ASCEND‐only sites and two of the seven dual sites began intervention or added their second intervention in March 2020 or later.

### 2.5 Outcome Measure

For each clinic and each study month, we measured screening rates as the number of documented social risk screenings per 100 distinct adult patients seen within the clinic that month.

### 2.6 Baseline Covariates

Clinics participating in ASCEND or AHC were not randomly selected (only the timing of implementation support start date was randomized along ASCEND clinics due to the step wedge design). To provide better balance in observed covariates between groups, we utilized clinic and patient population characteristics for matching nonparticipating clinics to the control pool of clinics. These covariates included aggregate measures obtained through EHR data from a 6‐month baseline period, with additional fixed characteristics. Aggregate measures included total ambulatory and telehealth visits (excluding COVID‐related visits), distinct number of patients seen, and percentages of patients aged 18 years or older, female, with public insurance, uninsured, patient self‐selected Hispanic or non‐Hispanic Black, and who preferred a non‐English language. OCHIN clinics follow the Office of Management Budget standards for race and ethnicity by collecting these two variables separately. Additionally, clinics' baseline social risk screening rates, years of operation at study start, presence of a designated primary care department (details for this covariate included in Section 2.7), having co‐located social services, Federally Qualified Health Center status, urban/rural location, and Medicaid expansion state status were included.

### 2.7 Control Matching

Using OCHIN EHR data we identified 211 sites eligible to serve as controls. To be eligible, sites must have operated outside any CHC organization containing clinics participating in either ASCEND or AHC, the clinic opened for business at least 6 months prior to September 2018 (first intervention month), and remained open during the full study period. Additionally, to align with ASCEND and AHC recruitment criteria, we limited the potential control pool to sites that had ever documented social risk screening. Utilizing R's *MatchIt* [23] package we employed nearest‐neighbor matching without replacement from a generalized linear model that regressed intervention status on the baseline covariates described above. An absolute standardized mean difference between intervention and control site of less than 0.2 indicated good balance [24]. Only the baseline indicator for a clinic having a designated primary care department did not reach this balance (Figure S1); this indicator does not suggest the clinic did not provide primary care, simply that it did not house a distinct department with this designation. We included this covariate in our CS‐DID modeling to ensure doubly robust estimates of treatment effect by controlling for residual confounding.

### 2.8 Statistical Analysis

We describe clinic characteristics summarizing baseline covariates overall and by intervention and control status. To estimate the effect of the interventions on social risk screening, we utilized the CS‐DID approach to four comparisons as follows: (1) Clinics receiving any intervention versus clinics receiving none; (2) clinics receiving financial incentives first and implementation support later versus clinics only receiving financial incentives (the addition of implementation support to financial incentives); (3) clinics receiving implementation support first and financial incentives later versus clinics only receiving implementation support (the addition of financial support to implementation support); and (4) clinics receiving financial support versus clinic receiving none. Assessment of the impact of implementation support alone was conducted in the ASCEND study, a stepped‐wedge cluster randomized trial [19].

The CS‐DID ensures a more robust treatment effect estimate than standard DID when there are multiple group‐time starts resulting from a staggered treatment adoption [25]. Assuming heterogeneous treatment effects and the staggered implementation of interventions, a standard DID approach would likely yield biased estimations of the average treatment effect from violation of the parallel trends assumption. The CS‐DID method corrects this violation through stabilized propensity scoring and average treatment effect estimation for each group‐time combination. In this case, 13 separate DID estimates are produced and then aggregated for an overall treatment effect estimate. For these analyses, only clinics never treated served as controls for all two‐by‐two DID estimations. We report the average treatment effect on the treated with 95% confidence intervals for the full study period. Additionally, through linear combinations of dynamic treatment effects, we report effects separately for the 12‐, 18‐, and 24‐month blocks following intervention. These follow‐up time periods allowed for understanding of the intervention impacts during both the relatively higher incentive period ($10/screen during the first 12 months, and $2/screen during Months 13–36). We describe raw screening rate averages for the intervention and control group clinics collectively to aid interpretation of the effect size. Finally, we performed two sensitivity analyses. First, we performed a stacked DID in event study form. In contrast to standard CS‐DID which uses the most recent pre‐treatment period as the base period and inverse probability weighting together with regression adjustment, for the stacked event study we utilized the six most recent pretreatment periods and unweighted regression for a robustness and efficiency trade‐off test. Second, we repeated the CS‐DID approach using a *t* − 2 estimator instead of a *t* − 1 estimator to evaluate the robustness of the main model estimates to pre‐period outcome fluctuations. Data preprocessing was conducted in R version 4.3.0 and analyses carried out in Stata version 15.1 using two‐sided testing and set 5% type I error. The research was approved by the local Human Research Protection Program.

## 3 Results

Thirty‐two control clinics matched the full sample of 32 intervention clinics on the clinic and patient population characteristics shown in Table 1. The average social risk screening rate for clinics during the 6‐month baseline period was 2.8 screens for every 100 distinct patients seen (intervention group: 2.7 [SD = 5.0]; control group: 2.9 [SD = 10.5]). During this time, intervention clinics on average saw 2943 distinct patients, compared to 2686 in control clinics. The vast majority (89.1%) of clinics were in urban locations and almost all were in Medicaid expansion states (95.3%); 65.6% had onsite social services (one or more staff designated as: case management, social work, community health worker, panel manager/coordinator, or outreach worker). Among control clinics, we observed an overall secular trend during the 55‐month project period in which average monthly social risk screening increased from around one to six screens for every 100 distinct adult patients. Event study plots for all four analyses support the assumption of pre‐period parallel trends (Figures S2–S5).

**TABLE 1:** Clinic and patient population characteristics for the full sample.

	Control (*N* = 32)	Intervention (*N* = 32)	Total (*N* = 64)
Urban locale	29 (90.6%)	28 (87.5%)	57 (89.1%)
FQHC	22 (68.8%)	22 (68.8%)	44 (68.8%)
County health department	10 (31.3%)	7 (21.9%)	17 (26.6%)
Primary care department^a^	29 (90.6%)	31 (96.9%)	60 (93.8%)
Social services on site	20 (62.5%)	22 (68.8%)	42 (65.6%)
Years active September 2018			
< 2	6 (18.8%)	6 (18.8%)	12 (18.8%)
2–4	8 (25.0%)	8 (25.0%)	16 (25.0%)
5+	18 (56.3%)	18 (56.3%)	36 (56.3%)
Expansion state	31 (96.9%)	30 (93.8%)	61 (95.3%)
Summary statistics for March through September 2018, mean (standard deviation)			
Distinct patients	2686.3 (2591.9)	2942.8 (3122.7)	2814.6 (2849.7)
Encounters	8114.0 (8148.2)	8982.8 (10250.9)	8548.4 (9196.1)
SDH screening rate			
Per 100 distinct patients	2.9 (10.5)	2.7 (5.0)	2.8 (8.1)
% Female	61.6 (12.0)	59.0 (14.1)	60.3 (13.1)
% Private insurance	17.9 (16.3)	18.3 (12.2)	18.1 (14.3)
% Public insurance	59.8 (17.9)	56.4 (14.4)	58.1 (16.2)
% Uninsured	22.3 (14.1)	25.3 (14.8)	23.8 (14.4)
% Hispanic	25.6 (21.3)	23.9 (21.9)	24.8 (21.4)
% Non‐Hispanic Black	8.1 (15.0)	8.5 (18.2)	8.3 (16.6)
% Non‐Hispanic White	51.5 (26.8)	51.7 (26.0)	51.6 (26.2)
% Non‐English	22.8 (19.0)	23.8 (25.6)	23.3 (22.4)

*Note*: Location in States (*n*): Control clinics CA (10), MA (3), OH (1), OR (16), WA (1), WI (1) and Intervention clinics CA (1), GA (1), MA (1), MT (1), OH (6), OR (20), WA (1), WI (1).

Abbreviations: FQHC, federally qualified health center; SDH, social determinants of health.

^a^ Clinic has a designated primary care department on site.

### 3.1 The Impact of Financial Incentives and Practice Facilitation Implementation Supports on Social Risk Screening Rates

Clinics receiving any intervention (financial incentives through AHC and/or practice facilitation supports through ASCEND) on average screened more than control clinics for the first 12 months following the start of the intervention (DID per 100 patients: 4.11 [95% CI: 0.7, 8.16]). In other words, the increase in average monthly social risk screenings from pre‐ to post‐intervention was 4.11 patients (per 100 patients) greater in intervention clinics than it was in the control clinics for the first year following intervention. As seen in Table 2, this benefit of intervention was maintained and increased slightly when estimating treatment effect over the longer 18‐ and 24‐month post‐intervention periods. Intervention benefit was not sustained when examined across the full available time period (up to 55 months). Estimates derived using a *t* − 2 instead of a *t* − 1 estimator were essentially identical (Table S1). Point estimates and statistical significance obtained from the stacked DID sensitivity analysis were similar in direction and magnitude to the aforementioned CS‐DID results (Table S1). However, when examining the impact of financial incentives alone (versus no intervention) no estimates derived using CS‐DID (*t* − 1 or *t* − 2 estimator) achieved statistical significance while the estimates of the stacked DID did reach significance.

**TABLE 2:** Difference‐in‐difference estimates of social risk screening intervention treatment effects.

	Average treatment effect on the treated			
	Difference‐in‐difference per 100 patients (95% CI)			
	12 months	18 months	24 months	All 55 months
Intervention comparisons	Post‐intervention	Post‐intervention	Post‐intervention	Post‐intervention
Financial incentive and/or implementation support vs. none	4.11 (0.7, 8.16)^a^	4.48 (0.80, 8.15)^a^	4.66 (0.89, 8.43)^a^	3.32 (−0.43, 7.07)
Addition of implementation support to financial incentive	3.70 (0.34, 7.07)^a^	4.43 (−0.01, 8.87)^a^	4.18 (−0.57, 8.94)	4.74 (−0.94, 10.41)
Addition of financial incentive to implementation support	10.33 (−2.29, 22.94)	9.68 (−2.72, 22.09)	8.92 (−2.27, 20.12)	4.55 (−1.82, 10.92)
Financial incentive vs. none	6.13 (−7.52, 19.78)	5.57 (−4.81, 15.95)	4.86 (−3.66, 13.38)	1.86 (−4.75, 8.46)

*Note*: Estimates derived using Callaway and Sant'Anna difference‐in‐difference.

^a^ Statistical significance at *p*‐value ≤ 0.05. In this staggered intervention natural experiment all intervention clinics had at minimum 18 months of post‐intervention follow‐up; the earliest intervention clinics provided a maximum of 55 months post‐intervention follow‐up.

### 3.2 The Additive Impact of Financial Incentives and Practice Facilitation Implementation Supports on Social Risk Screening Rates

Among clinics participating in both AHC and ASCEND, the addition of the second intervention resulted in overall increased social risk screenings. The additional impact of ASCEND practice facilitation implementation support to the pre‐existing AHC financial incentives resulted in increased social risk screening in the 12 months post‐intervention (DID per 100 patients: 3.79 [95% CI: 0.34, 7.07]). This means an additional 3.79 patients (per 100) were screened per month for the first 12 months following intervention. This effect was sustained and strengthened at 18 months but did not last beyond 18 months post‐intervention. Examining the interventions additively in the other order of sequencing, the addition of AHC financial incentives to ASCEND practice facilitation supports resulted in a larger effect size but did not reach statistical significance at any post‐intervention time point (e.g., DID per 100 patients: 10.33 [−2.29, 22.94]). Similar results were observed using a *t* − 2 instead of *t* − 1 estimator (Table S1). In sensitivity analyses using stacked DID, for both practice facilitation and financial incentives additions, all point estimates were similar in 12‐ and 18‐month post‐intervention treatment effect estimations. However, the effect estimates for the addition of financial incentives reached statistical significance and those for the addition of practice facilitation did not (Table S1).

Given that AHC financial supports in the Oregon AHC demonstration site were limited to in‐state clinics, we also conducted sensitivity analyses limited to clinics in Oregon (20 intervention, 20 control clinics) and observed a nearly identical pattern of findings to that found when utilizing the full sample (see Table S2).

## 4 Discussion

New national and state policies related to the collection and reporting of social data have increased the urgency for evidence on strategies that facilitate the implementation and scaling of social risk screening, particularly in the care settings serving patients who are most likely to benefit. This study is the first to compare the impacts of two implementation support approaches that are common in primary care (tailored practice facilitation vs. financial incentives) on social risk screening rates. Considering the secular trend among control clinics (increase from one to six screens per 100 patients over the course of the study) and the DID estimates, receiving any intervention versus none, and the additional layering of practice facilitation support on top of financial incentives—approximately doubled CHCs' monthly social risk screening rates for the first 18 months following intervention. Our findings are consistent with those from the ASCEND trial, in which social risk screening was 2.45 times higher during the 6‐month tailored implementation support period and 2.16 times higher after the support period compared to the preintervention period [19]. Results further suggest that financial incentives may have a similar impact on increasing social risk screening that may be greatest if introduced after tailored practice facilitation is provided. Despite the statistically significant positive impacts of either and both financial incentives and practice facilitation supports, the practical significance of our findings is unclear. Given the very low baseline levels of screening documentation in study clinics, a doubling in screening activity among intervention clinics still meant these clinics were only screening four to five more patients (per 100 patients) per month than nonintervention sites following each clinic‐level intervention.

How might these findings inform future social care policymaking? First, it is relevant that study CHCs had very low baseline rates of screening documentation, so doubling screening is not impressive. The CMS 2024 social care quality measures for inpatient settings start with percent of patients screened for social risks annually [26]; if and when these measures are extended to primary care settings (which CMS indicates is planned), CHCs are likely to need robust implementation supports to meet goals. The results presented here suggest that both monetary financial incentives and practice facilitation supports might help somewhat, but that far more support than was provided to any of these study clinics may be necessary to attain screening goals.

The low baseline rates of social risk screening documentation seen here also may stem from a more foundational problem regarding structured social risk screening in CHC settings. In qualitative research published previously, CHC clinic staff noted that standardized screening/documentation practices were often experienced as undermining more relational, team‐based social care [27]. As a consequence, structured screening tools were often adapted or abandoned to preserve the perceived benefits of conversation‐based, individual‐tailored social care. A related concern reported in our own and other's work is that standardized social risk screening tools often include social risk domains for which there are inadequate social services (e.g., housing), and some clinic staff are uncomfortable conducting assessments without being able to provide resources in response to reported need [27, 28, 29, 30]. That might mean that national policies' emergent definitions of high quality social care are not aligned with CHC teams' lived definitions [27].

Second, social care policymaking should reflect lessons from nonsocial care‐specific practice change efforts. Implementation supports—whether they are focused on financial incentives or practice facilitation—require strong infrastructure. The building blocks model of high‐performing primary care developed by Bodenheimer et al. 20 years ago underscores the importance of strong leadership buy‐in and support, robust quality improvement processes, and advanced team‐based care [31]. In the CHC environment, primary care workforce shortages, chronic underfunding, and staff turnover and burnout reduce the impact of all new practice change initiatives, including those focused on social care [32]. Hence the modest financial incentives and practice facilitation supports included in the AHC and ASCEND were always unlikely to overcome the more profound barriers to any form of practice change in CHC settings.

It is especially intriguing that financial reimbursements only modestly increased social risk screening rates given the investments in financial reimbursements at the national policy level. The direction and magnitude of the effects was similar across the primary and sensitivity analyses, though only reached statistical significance in the latter. The relatively minor impact of financial incentives might be explained not only by the factors above, but also in part by parallel qualitative work conducted with clinics in this sample and described elsewhere [33]. In those interviews, clinicians, frontline staff, and administrative leaders from Oregon CHCs did not think small financial incentives strongly influenced the behaviors of staff responsible for conducting screening. Respondents noted that for financial incentives to substantively change CHC screening practices, not only would staff need to be made aware of the incentives, but the incentives would need to be sizable enough to expand the clinic's social care workforce [33]. Nonetheless, it is noteworthy that in the primary analyses, the largest DID estimates were documented after the addition of financial incentives to practice facilitation implementation supports. This may suggest that clinics are better able to benefit from small monetary incentives after practice facilitation supports have already helped them assign staff responsibilities and clear workflows.

Finally, in the Oregon AHC demonstration, financial incentives were tested at a relatively modest reimbursement rate ($10/screen for the first year followed by $2/screen). We cannot predict whether a larger reimbursement rate or direct payments to patients—both suggestions from participants in our qualitative work [33]—might have larger impacts on screening rates. Future research might leverage the range of national and state initiatives to compare different incentive amounts and/or different beneficiaries for those incentives (e.g., clinic vs. patient) to better inform policymaking in this area. Certainly policymakers should at least consider that providing financial incentives on their own may be necessary but inadequate to facilitate the implementation and maintenance of social risk screening in CHCs.

Several limitations should be considered when interpreting these findings. First, we could not control for a number of factors that co‐occurred during the nearly 5‐year study period. These included the COVID‐19 pandemic, which impacted CHC social risk screening in nonuniform ways, and the implementation support efforts that could have co‐occurred locally or regionally. However, time‐varying effects, including the timing of intervention starts and subsequent varying pre‐periods, are captured in our statistical approach and reduce our concerns about the effects of these factors. Second, results are limited to social risk screening that was documented in a specific section of the EHR, which undercounts social risk screening activities by clinical staff that are not documented or are documented elsewhere. That said, the focus of both AHC and ASCEND was on increasing documented social risk information in EHRs; this aligns with the federal policies that will require EHR documentation [26]. Finally, since current CMS quality metrics focus on screening rates, we elected to examine that outcome. Yet social risk screening ideally yields interventions that benefit patient health and wellbeing. Future studies should examine the range of implementation supports needed to increase a wide range of meaningful outcomes, including social service referrals and connections, patients' experiences of care, and health outcomes. There are also likely important differences that were not accounted for between clinics that participate in social screening interventions (ASCEND and AHC) and those that do not. Although we attempted to control for patient and clinic confounders, selection bias may remain. Last, while prior research suggests that in some instances matching on pre‐period data may introduce bias [34], we chose to match control clinics to the full set of intervention clinics (without trimming intervention clinics) utilizing pre‐period outcome data. This was done for pragmatic (to compare clinics engaged in a similar amount of social risk screening prior to implementation support) and conceptual reasons (dynamic start times, CS‐DID method assumptions and use of last pre‐period time point). While this introduced the possibility of bias, we felt the benefits of this approach outweighed potential disadvantages.

## 5 Conclusion

As social risk screening becomes increasingly tied to policy and payment, it is critical to identify strategies that can support clinics' ability to implement this practice. This is particularly true for CHCs, which disproportionately serve patients experiencing social/economic barriers to health. The financial incentives and practice facilitation supports provided in these interventions individually and combined contributed to relatively modest changes in the numbers of CHC patients screened. Supports focused on social care will likely need to be coupled with foundational investments in the primary care safety net's quality improvement infrastructure to achieve and sustain universal screening.

## Conflicts of Interest

The authors declare no conflicts of interest.

## Supporting information


**Data S1.** Supporting Information.
