# Supplementary material for: The Combined and Comparative Impacts of Financial Incentives Versus Practice Facilitation Implementation Support for Social Risk Screening in Community Health Centers
Source: Health Serv Res. 2025 Feb 10;60(Suppl 3):e14448. doi: 10.1111/1475-6773.14448 (PMC12052507; doi:10.1111/1475-6773.14448)
Supplement: Supplementary file 1 — Data S1. Supporting Information. [file HESR-60-0-s001.docx]

**Appendix Figure 1.** Balance plot showing Absolute Standardized Mean Difference (ASMD) for variables used in 1:1 Nearest Neighbor matching, before and after matching. Matching on propensity scores generated with generalized linear model on intervention status. Matched 32 (of 211) eligible controls to 32 intervention clinics.


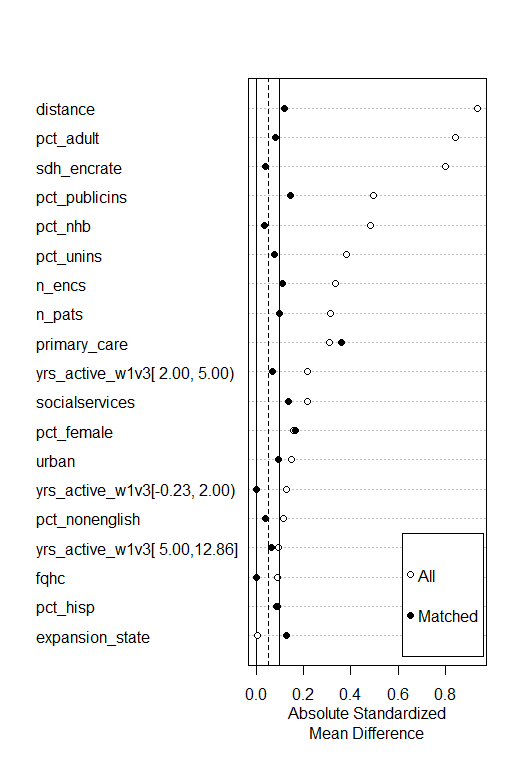


Distance

% Adult patients

SDH screening rate

% Public Insurance

% Non-Hispanic Black

% Uninsured

Encounters

Distinct Patients

Primary Care Department

Years Active 2-5

Social Services on Site

% Female

Urban Locale

Years Active < 2

% Non-English

Years active 5+

FQHC

% Hispanic

Expansion State

**Appendix Figure 2.** Event Plot Comparing Financial Incentive and/or Implementation Support vs. Neither.


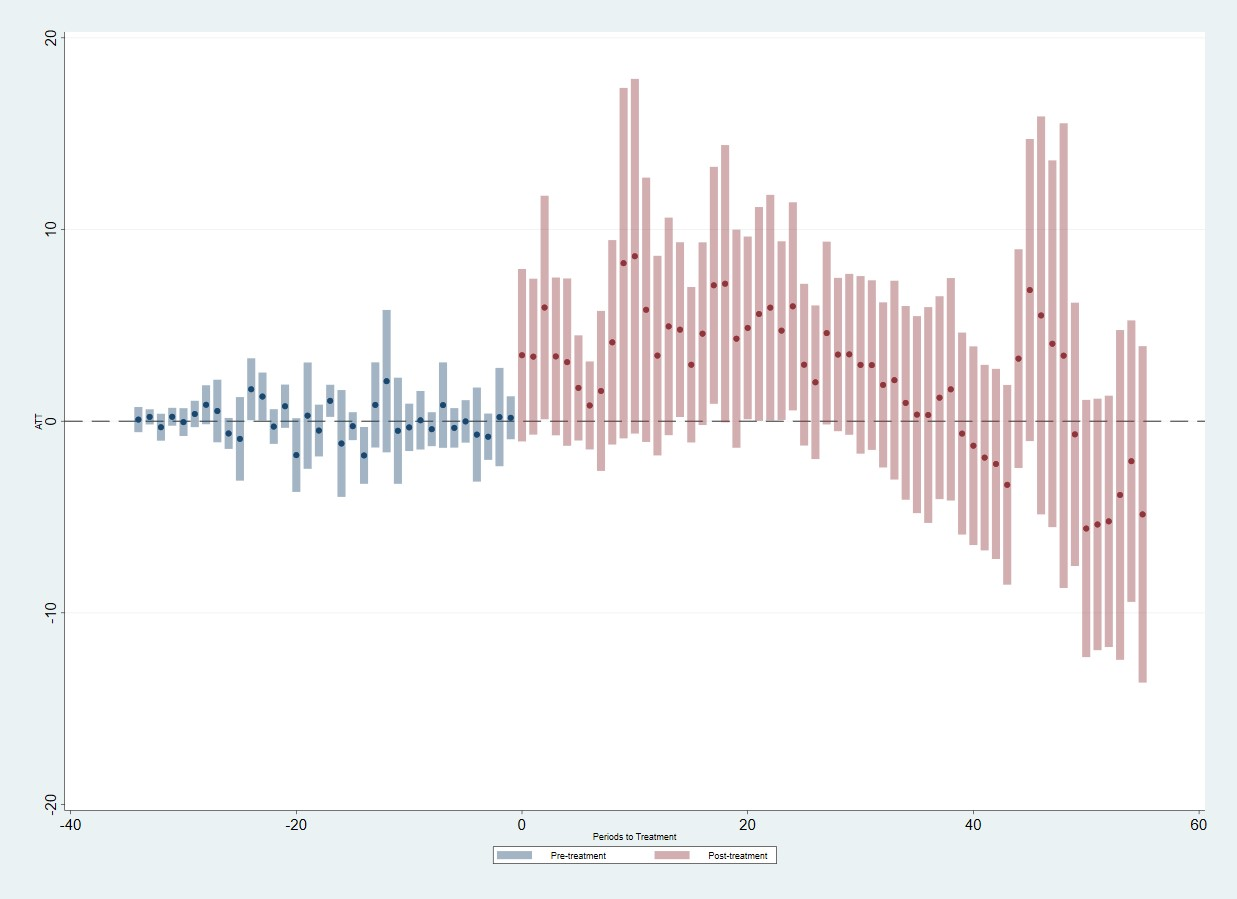


**Appendix Figure 3.** Event Plot Comparing the Addition of Implementation Support to Financial Incentive Alone.

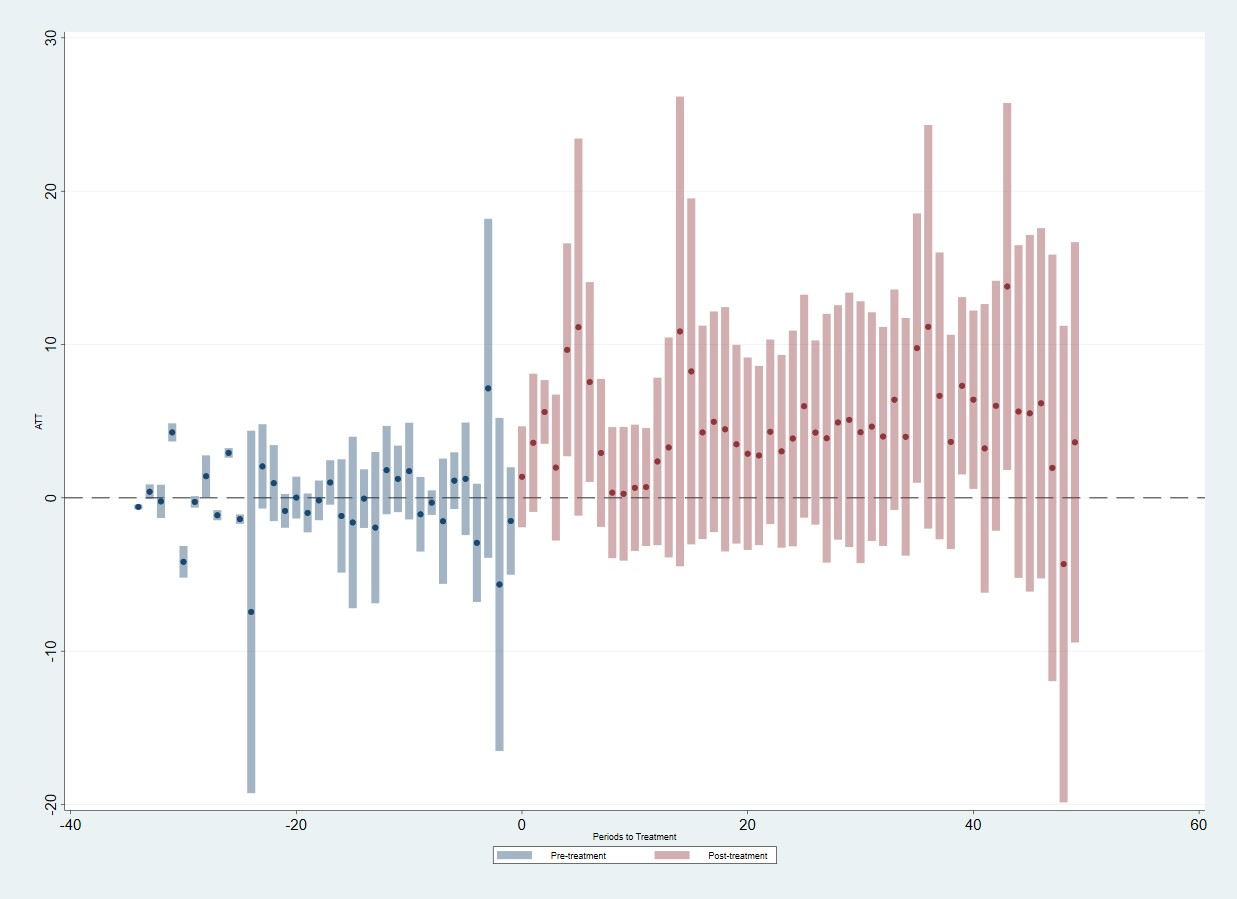


**Appendix Figure 4.** Event Plot Comparing the Addition of Financial Incentive to Implementation Support Alone.


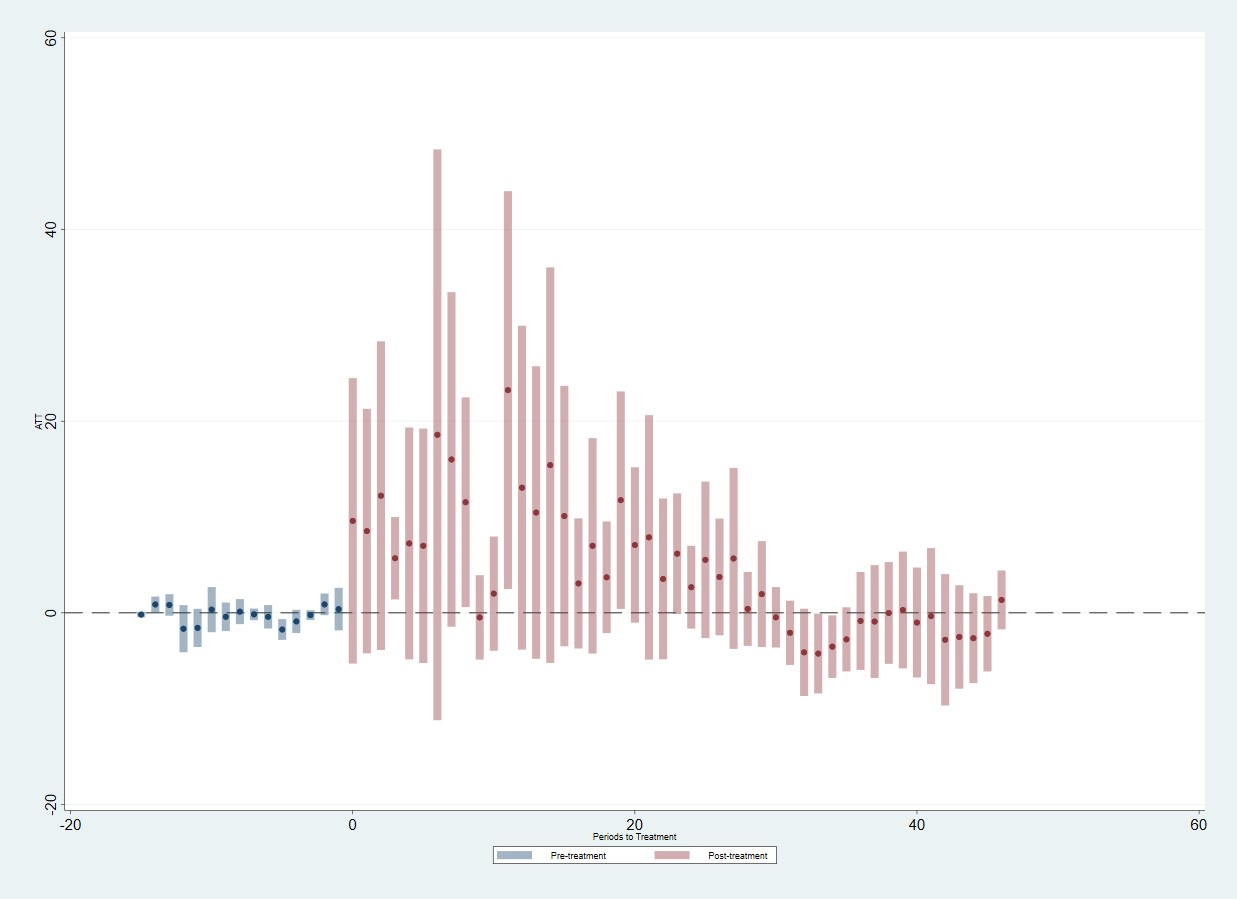


**Appendix Figure 5.** Event Plot Comparing Financial Incentive to No Intervention.

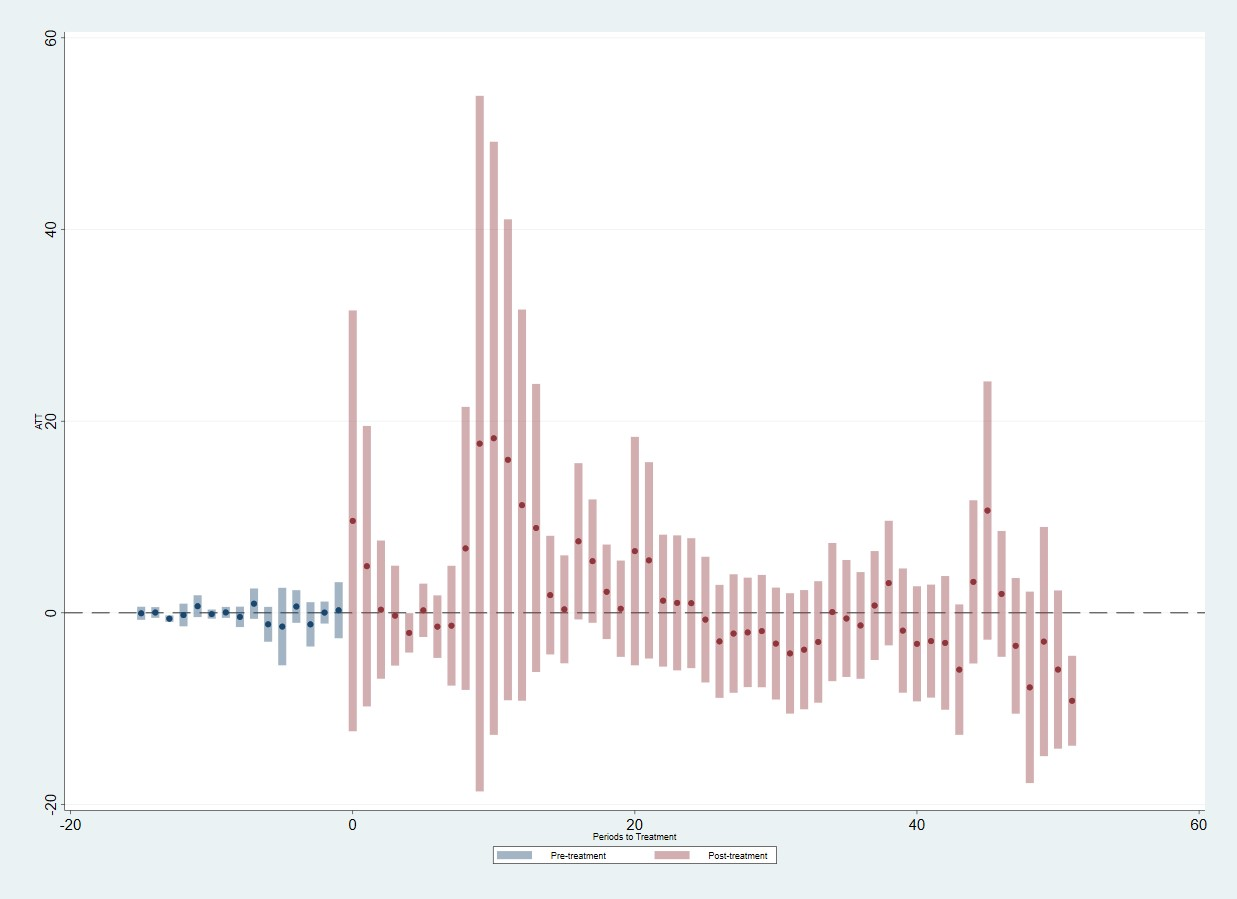


**Appendix Table 1.** Difference-in-difference estimates of social risk screening intervention treatment effects for CS-DID t-1, CS-DID t-2, and Stacked DID.

|  | **Average Treatment Effect on the Treated,** | | | | |  |  |  |
| --- | --- | --- | --- | --- | --- | --- | --- | --- |
|  | **Difference-in-Difference per 100 Patients (95% CI)** | | | | |  |  |  |
|  | *12 Months* |  | *18 Months* |  | *24 Months* |  | *All 55 Months* |  |
| **Intervention Comparisons** | *Post-Intervention* | *p-value* | *Post-Intervention* | *p-value* | *Post-Intervention* | *p-value* | *Post-Intervention* | *p-value* |
| Financial Incentive and/or Implementation Support vs. None |  |  |  |  |  |  |  |  |
| CS-DID t-1 Estimator | 4.11 (0.7, 8.16)* | 0.046 | 4.48 (0.80, 8.15)* | 0.017 | 4.66 (0.89, 8.43)* | 0.015 | 3.32 (-0.43, 7.07) | 0.082 |
| CS-DID t-2 Estimator | 4.42 (0.41, 8.43)* | 0.031 | 4.75 (1.13, 8.36)* | 0.01 | 4.91 (1.22, 8.60)* | 0.009 | 3.62 (-0.08, 7.34) | 0.055 |
| Stacked DID | 2.34 (1.51, 3.18)* | <0.001 | 2.64 (1.77, 3.50)* | <0.001 | n/a |  | n/a |  |
| Addition of Implementation Support to Financial Incentive |  |  |  |  |  |  |  |  |
| CS-DID t-1 Estimator | 3.70 (0.34, 7.07)* | 0.031 | 4.43 (-0.01, 8.87)* | 0.050 | 4.18 (-0.57, 8.94) | 0.085 | 4.74 (-0.94, 10.41) | 0.102 |
| CS-DID t-2 Estimator | 3.54 (0.22, 6.85)* | 0.037 | 4.27 (-0.23, 8.76) | 0.063 | 4.02 (-0.79, 8.82) | 0.102 | 4.58 (-1.15, 10.30) | 0.117 |
| Stacked DID | 1.59 (-3.14, 6.32) | 0.509 | 2.21 (-2.00, 6.41) | 0.303 | n/a |  | n/a |  |
| Addition of Financial Incentive to Implementation Support |  |  |  |  |  |  |  |  |
| CS-DID t-1 Estimator | 10.33 (-2.29, 22.94) | 0.109 | 9.68 (-2.72, 22.09) | 0.126 | 8.92 (-2.27, 20.12) | 0.118 | 4.55 (-1.82, 10.92) | 0.162 |
| CS-DID t-2 Estimator | 10.78 (-2.63, 24.20) | 0.115 | 10.14 (-3.10, 23.35) | 0.132 | 9.38 (-2.61, 21.36) | 0.125 | 5.00 (-2.11, 12.11) | 0.168 |
| Stacked DID | 10.11 (7.42, 12.80)* | <0.001 | 9.97 (7.44, 12.49)* | <0.001 | n/a |  | n/a |  |
| Financial Incentive vs. None |  |  |  |  |  |  |  |  |
| CS-DID t-1 Estimator | 6.13 (-7.52, 19.78) | 0.379 | 5.57 (-4.81, 15.95) | 0.293 | 4.86 (-3.66, 13.38) | 0.264 | 1.86 (-4.75, 8.46) | 0.582 |
| CS-DID t-2 Estimator | 7.57 (-5.86, 21.00) | 0.269 | 7.01 (-3.23, 17.24) | 0.180 | 6.30 (-2.21, 14.80) | 0.147 | 3.18 (-3.58, 9.94) | 0.356 |
| Stacked DID | 3.23 (1.35, 5.11)* | 0.001 | 3.11 (1.18, 5.04)* | 0.002 | n/a |  | n/a |  |
| *CS, Callaway & Sant'Anna; DID, Difference-in-Difference;* | | | | | | | | |
| CSDID t-1 & t-2 estimators derived using the csdid package in Stata with standard errors clustered at the clinic (panel) level and doubly-robust inverse probability weighting adjusted for whether clinics provided social services or had designated primary care departments. Starred values (*) indicate statistical significance at p-value<=0.05. In this staggered intervention natural experiment all intervention clinics had at minimum 18 months of post-intervention follow-up; the earliest intervention clinics provided a maximum of 55 months post-intervention follow-up. | | | | | | | | |
| Stacked DID estimates derived using the stackdid package in Stata with fixed effects for group-time, clinic, and whether clinics provided social services or had designated primary care departments. Only clinics never treated served as controls. In event study form, a 6-month pre-period was used to estimates differences at 12 and 18 month post-intervention. Starred values (*) indicate statistical significance at p-value<=0.05. | | | | | | | | |

**Appendix Table 2.** Difference-in-difference estimates of social risk screening intervention treatment effects limited to Oregon clinics.

|  | **Average Treatment Effect on the Treated,** | | |  |
| --- | --- | --- | --- | --- |
|  | **Difference-in-Difference per 100 Patients (95% CI)** | | |  |
|  | 12 Months | 18 Months | 24 Months | All 55 Months |
| **Intervention Comparisons** | Post-Intervention | Post-Intervention | Post-Intervention | Post-Intervention |
| Financial Incentive and/or Implementation Support vs. None | 5.19 (0.6, 9.79)* | 4.35 (0.4, 8.30)* | 4.11 (0.17, 8.05)* | 2.55 (-0.38, 5.47) |
|  |  |  |  |  |
| Addition of Implementation Support to Financial Incentive | 3.70 (0.34, 7.07)* | 4.43 (-0.01, 8.87)* | 4.18 (-0.57, 8.94) | 4.74 (-0.94, 10.41) |
|  |  |  |  |  |
| Addition of Financial Incentive to Implementation Support | 9.02 (-2.43, 20.47) | 8.33 (-3.20, 19.86) | 7.41 (-2.88, 17.71) | 4.50 (-1.15, 10.17) |
|  |  |  |  |  |
| Financial Incentive vs. None | 5.79 (-6.31, 17.89) | 4.01 (-5.09, 13.11) | 2.35 (-4.69, 9.39) | -0.18 (-4.67, 4.31) |
| Estimates derived using Callaway & Sant'Anna Difference-in-Difference. Starred values (*) indicate statistical significance at p-value<=0.05. In this staggered intervention natural experiment all intervention clinics had at minimum 18 months of post-intervention follow-up; the earliest intervention clinics provided a maximum of 55 months post-intervention follow-up. For the Addition of Implementation Support to Financial Incentive result estimates are the same in the full sample because all AHC-only and Dual clinics are only located in Oregon. | | | | |
